# *NSF* gene variants cause developmental and epileptic encephalopathy 96: expanding genotype and phenotypic spectrum with prenatal-onset features

**DOI:** 10.3389/fgene.2026.1882449

**Published:** 2026-08-04

**Authors:** Qi Yang, Zailong Qin, Jiao Li, Qiang Zhang, Xunzhao Zhou, Sheng Yi, Shujie Zhang, Weiliang Lu, Shang Yi, Sheng He, Jingsi Luo

**Affiliations:** 1 Guangxi Key Laboratory of Birth Defects Research and Prevention, Guangxi Key Laboratory of Reproductive Health and Birth Defects Prevention, Maternal and Child Health Hospital of Guangxi Zhuang Autonomous Region, Nanning, China; 2 Department of Genetic and Metabolic Central Laboratory, Maternal and Child Health Hospital of Guangxi Zhuang Autonomous Region, Nanning, China; 3 Guangxi Clinical Research Center for Birth Defects, Maternal and Child Health Hospital of Guangxi Zhuang Autonomous Region, Nanning, China; 4 Prenatal Diagnosis Center, Maternal and Child Health Hospital of Guangxi Zhuang Autonomous Region, Nanning, China; 5 Guangxi Clinical Research Center for Pediatric Diseases, Maternal and Child Health Hospital of Guangxi Zhuang Autonomous Region, Nanning, China

**Keywords:** *de novo*, developmental and epileptic encephalopathy 96, novel variant, NSF, whole exome sequencing

## Abstract

**Background:**

Developmental and epileptic encephalopathy 96 (DEE96, OMIM 619340) is a rare autosomal dominant disorder caused by heterozygous variants in the *NSF* gene, encoding a key AAA + ATPase involved in SNARE-mediated membrane fusion. To date, only four pathogenic variants have been reported.

**Methods:**

Trio whole-exome-sequencing and Sanger validation were performed in a fetus presenting with multiple prenatal anomalies. Bioinformatic analyses and literature review were conducted to characterize genotype-phenotype correlations.

**Results:**

We identified a novel *de novo* heterozygous missense variant [c.1055 A>G; p (Asn352Ser)] in the D1 domain of NSF, classified as likely pathogenic per ACMG/AMP criteria. Prenatal ultrasound revealed increased nuchal fold thickness, left clubfoot, severe anemia with cardiac enlargement, and hepatosplenomegaly. Increased nuchal fold thickness and left clubfoot had not been previously documented in DEE96 cases. This represents the second prenatal diagnosis and fifth case overall.

**Conclusion:**

Our findings expand the genotypic and phenotypic spectrum of DEE96, demonstrating that DEE96 manifests as a multisystem disorder with prenatal onset. The high incidence of severe anemia suggests hematological involvement may be a characteristic feature. These observations support considering NSF variants in the differential diagnosis of complex fetal syndromes with neurological and hematological abnormalities.

## Background

Next-generation sequencing (NGS) has been instrumental in the diagnosis of rare genetic diseases, by virtue of its capacity to facilitate high-throughput analysis of genomic variants with exceptional speed and accuracy. Since its first successful application for Mendelian disorder gene identification in 2010, next-generation sequencing (NGS) has become the cornerstone of molecular diagnostics, significantly improving the diagnostic yield for patients with rare and genetically heterogeneous conditions (Ng et al., 2010; Boycott et al., 2013). Whole exome sequencing (WES) and whole genome sequencing (WGS) have been particularly instrumental in reducing the duration of the diagnostic process for neurodevelopmental disorders, as they permit the simultaneous interrogation of thousands of genes and the identification of novel disease-causing variants that would be overlooked by traditional Sanger sequencing (Yang et al., 2013; Tengsujaritkul et al., 2026). Recent studies have highlighted the diagnostic utility of NGS in uncovering pathogenic variants underlying cognitive impairment, microcephaly, and neurodevelopmental disorders with ataxia, further underscoring its clinical value in precision medicine (Yavas et al., 2026; Ercoskun et al., 2026; Baris et al., 2026).

NSF deficiency syndrome, also known as Developmental and epileptic encephalopathy 96 (DEE96) (OMIM 619340), is a very rare autosomal dominant disorder characterized by global developmental delay, failure to thrive, microcephaly, hypotonia, and epileptic encephalopathy (Suzuki et al., 2019; Hayashi et al., 2023; Li et al., 2024). It is caused by heterozygous variants in the NSF gene (NM_006178.4; OMIM 601633), locate at chromosome 17q21.31, which contains 21 exons and encodes the N-ethylmaleimide sensitive factor, vesicle fusing ATPase protein involved in membrane fusion (Rizo, 2018; Kh et al., 2022; Söllner et al., 1993). As a homohexameric AAA-type ATPase that mediates membrane fusion, NSF precisely drives vesicular transport underlying neurotransmitter release and hormone secretion through dynamic SNARE (soluble NSF attachment protein receptor) complex assembly/disassembly orchestrated by calcium signaling (Ferro-Novick and Brose, 2013). The dysfunction of this machinery is increasingly recognized as the fundamental pathogenic basis for diabetes and neurological disorders. Pathogenic variants of SNARE complex components are associated with Alzheimer’s disease, Parkinsonism, autism, and psychiatric disorders (Ramakrishnan et al., 2012). However, monogenic disease phenotypes of NSF mutations had not been described until recently.

To date, nine individuals carrying pathogenic or likely pathogenic variants in the NSF gene have been identified. However, only four of these individuals have been described in detail as exhibiting the clinical features of DEE96 (Supplementary Table S1) (Suzuki et al., 2019; Hayashi et al., 2023; Li et al., 2024). All variants identified were missense variants predicted to defective autophagy through overactivation of the mammalian/mechanistic target of rapamycin (mTOR) pathway. Further reports on NSF variants and their phenotypes will be crucial for understanding this disease. Here, we investigated a previously unreported heterozygous missense variant [c.1055 A>G; p (Asn352Ser)] in a Chinese foetus with DEE96. Additionally, we have summarised the genotypic, phenotypic and clinical features of DEE96 through a review of the literature.

## Materials and methods

### Subjects and ethics approval

The study was approved by the Institutional Review Board and Ethics Committee of the Guangxi Maternal and Child Health Hospital. Detailed informed consent was obtained from the patient’s parents for the publication of their relevant clinical data in this paper.

### Prenatal ultrasonography

Ultrasound examinations were performed using a Toshiba Aplio 500 system (Toshiba Medical Systems, Tokyo, Japan) equipped with a 3–6 MHz transabdominal transducer, with imaging parameters standardized according to manufacturer protocols for obstetric scanning. The primary scans were conducted by an experienced fetal medicine specialist (>5 years of dedicated obstetric ultrasound experience). For quantitative parameters, two consultant sonographers independently acquired measurements, with each observer obtaining three consecutive readings to ensure reliability. The final values were calculated as the mean of these replicate measurements.

### Genetic analysis

Trio-based whole-exome sequencing (WES) was performed on the proband and both parents. Genomic DNA was extracted from amniotic fluid samples of the foetus using the QIAamp DNA Mini Kit (Qiagen, Hilden, Germany), while parental DNA was isolated from peripheral blood lymphocytes using the La-Aid DNA kit (Zeesan Biotech Co., Ltd., Xiamen, China). For each sample, 3 μg of DNA was sheared into fragments of 180–250 base pairs (bp), followed by end-repair, A-tailing, and adapter ligation. Exome capture was carried out using the Agilent SureSelect Human All Exon V6 Kit (Agilent Technologies, Santa Clara, CA, United States). The resulting libraries, with insert sizes ranging from 280 to 320 bp, were validated on a High-Sensitivity DNA chip using the 2,100 Bioanalyzer (Agilent Technologies). Subsequently, paired-end sequencing (150 bp) was performed on the Illumina HiSeq 2,500 platform (Illumina, San Diego, CA, United States). The raw reads were then aligned to the human reference genome GRCh38/hg38 using the Burrows-Wheeler Aligner (BWA, version 0.7.15). Variant calling and annotation were performed using TGex software (LifeMap Sciences Inc., version 5.7) and the Genome Analysis Toolkit (GATK). Prioritization of candidate variants was conducted using *in silico* pathogenicity prediction tools, including REVEL, CADD, and AlphaMissense. Sanger sequencing was performed to validate the candidate variant in the proband and available unaffected family members. Wild-type and mutant models were constructed using the AlphaFold server. The model with the highest Global Model Quality Estimation (GMQE) score was then selected for further analysis. The models were visualised using the PyMOL molecular graphics system (version 1.3, Schrödinger, LLC). The following primers, which were designed using Primer3, were used: 5′-CTT​CTC​ACC​AAA​ACT​CGG​C-3′ and 5′-CAT​CTT​TTT​CAC​CTG​GGT-3′ for c.1055 A>G; p (Asn352Ser). Variant interpretation followed the guidelines of the American College of Medical Genetics and Genomics/Association for Molecular Pathology (ACMG/AMP) (Richards et al., 2015).

## Result

**FIGURE 1:**
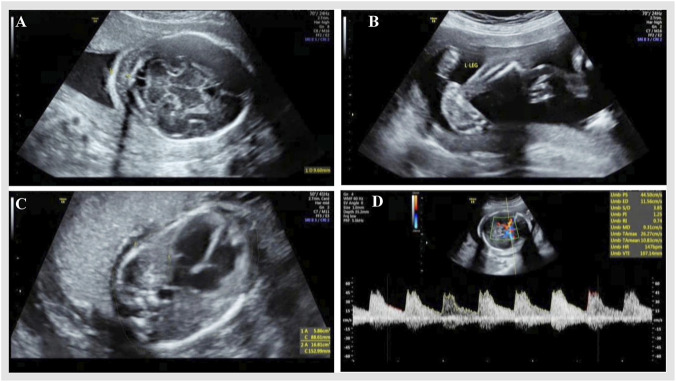
Phenotypes of the patients **(A–D)** Ultrasound images at 23 weeks’ gestation showing: **(A)** increased nuchal fold thickness (9.8 mm); **(B)** left clubfoot;**(C)** cardiomegaly with a cardiothoracic ratio of 0.35; **(D)** elevated middle cerebral artery peak systolic velocity (MCA-PSV: 44.5 cm/s).

A 31-year-old woman was referred to the Department of Prenatal Diagnosis and Genetic Counselling at the Guangxi Zhuang Autonomous Region Maternal and Child Healthcare Hospital in Nanning, China, because of left clubfoot and nuchal fold thickening. She conceived naturally and had a routine first-trimester ultrasound scan at 12 weeks of pregnancy, which revealed a nuchal translucency measurement of 1.6 mm. The parents were non-consanguineous and healthy, with no family history of neurological or other hereditary disorders. The mother denied drug exposure during the periconceptional period and pregnancy, and received routine antenatal care. At 23 weeks of gestation, detailed ultrasound examination revealed an appropriately grown fetus with increased nuchal fold (NF) thickness (9.8 mm), left clubfoot, cardiomegaly, hepatosplenomegaly, and elevated middle cerebral artery peak systolic velocity (MCA-PSV, 44.5 cm/s) (Figure 1). Upon follow-up at 31 weeks, these findings persisted, with additional sonographic evidence of fetal hepatosplenomegaly. Based on these combined Doppler and morphological abnormalities, fetal anemia was suspected. Cordocentesis confirmed severe anemia in the fetus (hemoglobin 54 g/L), with a red blood cell count of 1.45 × 10^12^/L. The fetal anemia was normocytic normochromic. The fetus has blood type AB and is Rh-positive. Maternal investigations revealed blood group AB Rh-positive with normal mean corpuscular volume (MCV); irregular antibody screening and infectious workup (syphilis, cytomegalovirus, and parvovirus B19) were all negative. No other significant structural anomalies were identified. After receiving genetic counselling, the parents opted to terminate the pregnancy at 32 weeks.

### Genetic analysis

**FIGURE 2:**
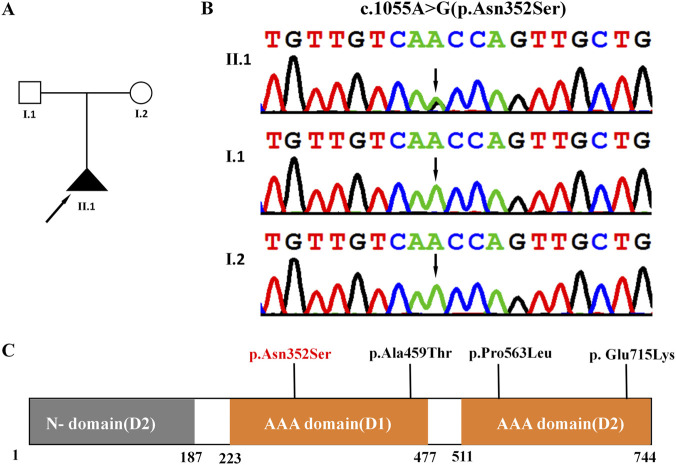
Genetic analysis of the affected proband **(A)** The spectrum of pathogenic variants in NSF. The red variants represent the novel variants identified in this study **(B)** Pedigree chart of the family of the fetus with developmental and epileptic encephalopathy 96. The proband is indicated by a black arrow **(C)** Sanger sequencing DNA chromatograms of NSF indicated the missense c.1055 A>G; p (Asn352Ser) variant was *de novo*.

**FIGURE 3:**
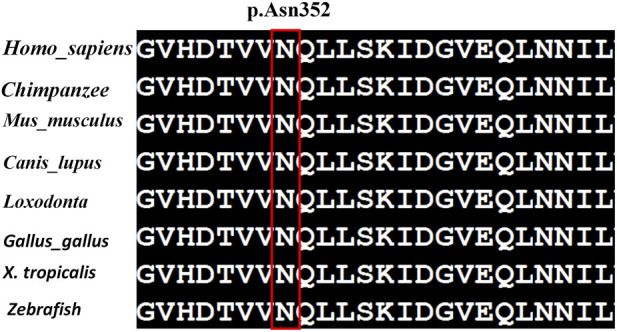
Multispecies alignment showing the strong conservation of NSF p. Asn352.

**FIGURE 4:**
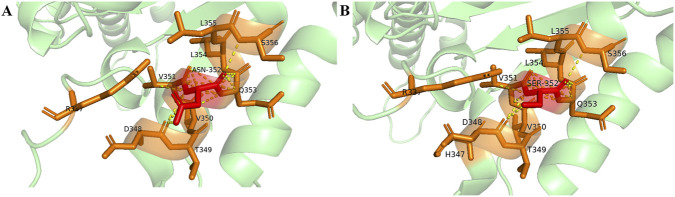
Predicted molecular basis of N352 S **(A)** Wild type **(B)** Mutant type. The Asn352Ser mutation causes the amino acid at position 352 to change from aspartic acid to serine, altering the orientation of the side chains near the mutation site and the potential hydrogen-bond interactions.

The proband underwent trio-based whole exome sequencing to investigate the genetic etiology of the multiple malformations (Figure 2A). The proband underwent trio-based whole exome sequencing to investigate the genetic etiology of the multiple malformations (Figure 2A). A *de novo* heterozygous missense variant [c.1055 A>G, p (Asn352Ser)] was identified in the NSF gene (Supplementary Figure S1). Sanger sequencing confirmed the presence of this variant in the proband and its absence in both parents, establishing its *de novo* origin (Figure 2B). Detailed analysis of the trio-WES data confirmed that the exon containing the variant had a sequencing depth of 244× and a variant allele fraction of 44.67%, with reads demonstrating high mapping quality (MAPQ) and balanced strand distribution; no significant strand bias or mapping artifacts were detected, confirming that this locus was adequately and uniquely mappably covered. Although the genomic region harboring NSF may present potential challenges for short-read sequencing, the variant-bearing exon does not fall within any known low-mappability or high-GC regions in the present dataset, and an IGV screenshot is provided in the supplementary materials to display read-level supporting evidence (including allele balance and read distribution) for direct visual assessment. The variant [c.1055 A>G; p (Asn352Ser)] in the *NSF* gene is novel and has not been previously reported. It is absent from the public population databases, including the Exome Sequencing Project, gnomAD (v4.1.1), the 1000 Genomes Project, and dbSNP. The variant is also not documented in disease-specific repositories such as ClinVar and the Human Gene Mutation Database. The p (Asn352Ser) variant is located within the D1 domain of the NSF hexamer, a region critical for ATP binding and hydrolysis, the essential driving force for SNARE complex disassembly (Figure 2C). Three complementary *in silico* approaches supported the pathogenicity of this variant: (i) REVEL (v1.3), an ensemble missense predictor (score: 0.73; pathogenic threshold >0.5); (ii) CADD (v1.6), which integrates conservation and functional annotation (PHRED-like score: 25.3; pathogenic threshold >20); and (iii) AlphaMissense, a structure-based predictor leveraging AlphaFold2 protein structures (score: 0.81; pathogenic threshold >0.564) (Table 1). Multiple sequence alignment showed that the Asn352 residue is highly conserved across diverse organisms (Figure 3). The Asn352Ser mutation causes the amino acid at position 352 to change from aspartic acid to serine, altering the orientation of the side chains near the mutation site and the potential hydrogen-bond interactions. A 3D protein structure visualisation analysis using PyMOL software revealed that the missense mutation c.1055 A>G (p.Asn352Ser) alters the orientation of side chains near the mutation site and potential hydrogen-bond interactions (Figure 4). According to the ACMG/AMP standards and guidelines, this variant is classified as ‘likely pathogenic’ based on the supporting criteria PS2, PM2 and PP3 (Table 1).

**TABLE 1:** Predicted pathogenicity of *de novo NSF* variant.

Gene	Variant (NM_145331.2)	Inheritance	Revel	AlphaMissense	CADD	ACMG/AMP
*NSF* (NM_006178.4)	c.1055A>G (p.Asn352Ser)	DNV	0.73	0.81	25.3	LP (PS2+PM2+PP3)

DNV, *de novo* variant; D, damaging; LP, Likely pathogenic.

## Discussion

Developmental and Epileptic Encephalopathy 96 (DEE96) is an emerging example of a ‘SNAREopathy’, which is a neurological disorder that arises from dysfunctional SNARE-mediated membrane fusion machinery (Boycott et al., 2013). Since its initial description, only three pathogenic variants [p (Ala459 Thr), p (Pro563Leu) and p (Glu715Lys)] have been reported in four patients who exhibited multi-domain developmental abnormalities, including global developmental delay, epileptic encephalopathy, hypotonia, feeding difficulties and severe anaemia (Suzuki et al., 2019; Hayashi et al., 2023; Li et al., 2024). Here, We describe a novel *de novo* heterozygous missense variant [c.1055 A>G; p (Asn352Ser)], which was identified prenatally. This is the second reported case of DEE96 diagnosed during the fetal period.

Clinically, the core postnatal phenotypes of DEE96 established by previous cases include global developmental delay, infantile-onset epileptic encephalopathy, microcephaly, hypotonia, and failure to thrive (Table 2) (Suzuki et al., 2019; Hayashi et al., 2023; Li et al., 2024). In contrast, prenatal ultrasound in this case revealed characteristic features including increased nuchal fold thickness, clubfoot, cardiomegaly, and hepatosplenomegaly, accompanied by severe fetal anemia, suggesting that neurological and extraneurological dysfunction may manifest during fetal development. To our knowledge, increased nuchal fold thickness and clubfoot are reported for the first time in association with DEE96. The left-sided clubfoot observed in this case likely reflects neuromuscular dysfunction secondary to impaired neurotransmitter release at the neuromuscular junction, a mechanism supported by findings that *NSF* variants can cause neuromuscular junction impairment (Hayashi et al., 2023). Furthermore, this case represents the third instance of severe anemia, bringing the total incidence of anemia to 60% (3/5), indicating that anemia may be a characteristic manifestation of DEE96. The anemia in these patients cannot be explained by common etiologies such as alloimmunization, infection, or α-thalassemia. The severe fetal anemia may reflect impaired erythrocyte membrane dynamics or defective autophagy in erythroid progenitors, given the essential role of autophagy in red blood cell maturation and mitochondrial clearance, suggesting that NSF may participate in hematopoietic processes or erythrocyte membrane dynamics regulation (Chen et al., 2008; Grosso et al., 2017). The hepatosplenomegaly may represent extramedullary hematopoiesis secondary to anemia, or alternatively, may indicate primary involvement of NSF in these tissues. These clinical findings further expand DEE96 from a purely neurodevelopmental disorder to a potentially systemic disease with prenatal onset, suggesting that NSF-related variants should be considered in the differential diagnosis of complex fetal syndromes associated with hematological abnormalities.

**TABLE 2:** Summary of reported information on clinical phenotypes associated with *NSF* variants in patients with DEE96.

Patients	Our patient	Suzuki et al. (Suzuki et al., 2019)		Hayashi et al. (Hayashi et al., 2023)	Li et al. (Li et al., 2024)	Total
Clinical data	Patient1	Patient2	Patient3	Patient4	Patient5	N = 5
Variants in NSF (NM_006178.4)	c.1055 A>G (p.Asn352Ser)	c.1375G>A (p.Ala459Thr)	c.1688C>T (p.Pro563Leu)	c.2143G>A (p.Glu715Lys)	c.1688C>T (p.Pro563Leu)	Missense = 4
Inherit	*De novo*	*De novo*	*De novo*	*De novo*	*De novo*	​
Affected exon/intron	10	13	15	19	15	​
Gender	NA	Femal	Femal	Femal	NA	​
Age at last examination	31-week-old fetus	36D (deceased)	6Y	4Y	17-week-old fetus	​
Birth week	NA	37 W	33 W	-	NA	​
Fetal hydrops	-	NA	+	-	+	2/4
Intrauterine growth restriction	-	NA	+	-	+	2/4
Anemia	Severe	-	+	-	Severe	3/5
Microcephaly	NA	-	<-3.4SD	-	NA	1/3
Respiratory problems	NA	+	+	-	NA	2/3
Intellectual disability	NA	NA	Severe	-	NA	1/2
Motor delay	NA	NA	Severe	-	NA	1/2
Vomiting	NA	+	NA	-	NA	1/2
Age at seizure onset	NA	Neonatal period	At birth	No seizure	NA	​
Seizure	NA	Tonic seizures	Myoclonic seizures	Mild spastic paraplegia and startle response (both only in infancy)	NA	3/3
EEG	NA	+	+	-	NA	2/3
Other features	Cardiomegaly, hepatosplenomegaly; increased middle cerebral artery peak systolic velocity (44.5 cm/s; >2.0 MOM),Nuchal fold thickening (14.4 mm),Left clubfoot	​	​	​	Cardiomegaly, hepatosplenomegaly; pleural effusion, ascites,increased middle cerebral artery peak systolic velocity (44.5 cm/s; >2.0 MOM),increased placental depth (2.7 cm)	​

DD96:Developmental and epileptic encephalopathy 96; Y: years; W: weeks; D:days; N: numbers; NA: not available; SD:standard deviation; EEG:Electroencephalography.

It is noteworthy that most mutations in *NSF* are lethal across species. The classic “comatose” (com) mutant in *Drosophila* is a temperature-sensitive paralytic mutant and was one of the earliest models to link NSF dysfunction to severe neurological phenotypes. At restrictive temperatures, this mutant exhibits rapid activity-dependent paralysis and synaptic transmission failure due to impaired vesicle recycling (Siddiqi and Benzer, 1976; Pallanck et al., 1995). Furthermore, biochemical analysis of the comt st53 mutation (S483L in dNSF1, corresponding to S491L in mammalian *NSF*) revealed a specific defect in α-SNAP-stimulated ATPase activity without impairing α-SNAP binding. When this mutation was engineered into yeast Sec18, it produced a dominant lethal phenotype *in vivo*, underscoring the exquisite sensitivity of the NSF hexamer to perturbations in ATPase regulation (Pallanck et al., 1995). Similarly, distinct temperature-sensitive comatose alleles that alter different amino acids within the D1 domain converge on a common phenotype of impaired SNARE complex disassembly and synaptic transmission failure, indicating that D1 domain integrity is essential for NSF function (Stewart et al., 2002). Consistent with these findings, the fetus in our study, which harbored a p. Asn352Ser mutation in the NSF D1 domain, exhibited a similarly severe phenotype. Multiple sequence alignment confirmed absolute conservation of this asparagine residue across diverse species. Analysis of the protein’s three-dimensional structure revealed that the c.1055 A>G missense mutation (p.Asn352Ser) altered the orientation of the side chains near the mutation site, affecting potential hydrogen-bond interactions. The NSF D1 domain is structurally arranged as a hexameric ATPase ring, and inter-subunit interactions are essential for coordinating ATP hydrolysis and substrate translocation (Moeller et al., 2012; Khan et al., 2025; Zhao and Brunger, 2016; Söllner et al., 1993). Recent cryo-EM studies have further demonstrated that NSF employs a sequential hydrolysis mechanism, whereby ATP binding and hydrolysis propagate around the D1 ring via specific inter-subunit interfaces, driving substrate threading and SNARE complex disassembly (Moeller et al., 2012; Khan et al., 2025; Zhao and Brunger, 2016; Söllner et al., 1993). Asn352 is located precisely at one such critical inter-subunit junction. Therefore, the p (Asn352Ser) substitution likely disrupts the structural integrity of the hexamer interface, potentially impairing either the assembly or stability of the NSF homohexamer or the allosteric communication required to coordinate ATP hydrolysis around the ring structure. Interestingly, one individual harbouring the p (Phe715Lys) mutation exhibited an unusually mild phenotype among previously reported patients (Hayashi et al., 2023). Structural analysis of NSF indicates that, in contrast to the P563L and A459 T mutants, the E715 K mutation is not located at a site that could interfere with the transmission of conformational changes in NSF monomers. Rather, it only partially obstructs the process by which NSF monomers undergo rotational ATP hydrolysis (Hayashi et al., 2023). This phenotypic heterogeneity reflects the mutation’s specific effects on different NSF functional domains, or its differential impact on the dynamics of tissue-specific SNARE complexes during development.

The pathogenic mechanisms underlying NSF deficiency syndrome remain incompletely elucidated. Current evidence indicates that disease-associated variants disrupt multiple interconnected cellular processes. As a homohexameric AAA + ATPase, NSF primarily orchestrates the disassembly of SNARE complexes following vesicle fusion, thereby facilitating SNARE protein recycling and maintaining subsequent membrane fusion events (Orr et al., 2017). This function is critical not only for regulating synaptic vesicle neurotransmitter release but also for autophagosome-lysosome fusion—a process essential for cellular homeostasis and neuronal survival (Ramakrishnan et al., 2012; Baudot et al., 2022). Moreover, beyond its canonical role in post-fusion SNARE recycling, biochemical and structural studies have revealed that NSF and its yeast orthologue Sec18 act as crucial priming factors, disassembling non-fusogenic binary SNARE complexes (e.g., syntaxin-1A–SNAP-25) prior to membrane fusion (Zhao et al., 2012). This pre-fusion disassembly prevents the formation of off-pathway SNARE assemblies and ensures the availability of fusion-competent SNAREs for trans-complex formation,a quality control function that is essential for synaptic vesicle priming (Söllner et al., 1993; Morgan, 1996; Coleman, 1974; Woodman, 1997). Recent atomic-resolution cryo-EM structures have directly observed the interaction between NSF and the binary syntaxin–SNAP-25 complex, revealing how the D1 ATPase ring drives its disassembly (White et al., 2018; White et al., 2025). In zebrafish, *NSF* mutations impair the disassembly of both binary and ternary SNARE complexes, leading to defects in the organizational structure and sensory function of myelinated axons. This provides physiological evidence in vertebrates that NSF-mediated preactivation is essential for normal neural development (Mo and Nicolson, 2011). Hayashi et al. (2023) further demonstrated that DEE-associated *NSF* variants cause defective autophagy through overactivation of the mTOR signaling pathway (Hayashi et al., 2023). In PC12 cells and patient-derived iPSC-differentiated neurons, pathogenic NSF variants led to aberrant autophagosome accumulation, impaired autophagic flux, and neurite degeneration characterized by beading morphology—these being hallmarks of neurodegeneration. Notably, treatment with the mTOR inhibitor rapamycin or overexpression of wild-type NSF significantly ameliorated these cellular phenotypes, establishing a causal link between NSF dysfunction, mTOR hyperactivation, and neurodegeneration (Hayashi et al., 2023). Further investigation suggested that aberrant mTOR activation in NSF-deficient cells may directly result from impaired autophagosome-lysosome fusion. Under physiological conditions, SNARE complexes mediate the fusion of autophagosomes with lysosomes to form autolysosomes for intracellular waste degradation, while NSF is responsible for recycling autophagy-related SNARE proteins (including STX17, SNAP29, and VAMP8) during this process (Ramakrishnan et al., 2012; Levine and Kroemer, 2019; Baudot et al., 2022). When NSF function is compromised, SNARE complex disassembly is obstructed, leading to autophagic flux arrest and potentially triggering compensatory mTORC1 activation as a cellular feedback response to lysosomal insufficiency (Saxton and Sabatini, 2017). This abnormal activation may establish a self-perpetuating vicious cycle: autophagy impairment promotes the accumulation of aberrant proteins and damaged organelles, further exacerbating cellular stress and ultimately driving neurodegenerative progression (Hayashi et al., 2023; Levine and Kroemer, 2019). The multi-system involvement observed in this case indicates that the impact of NSF dysfunction extends beyond the nervous system to affect fundamental cellular processes. Further functional studies are warranted to deepen our understanding of NSF-related disorders and their underlying mechanisms.

This study has several limitations. Firstly, the small number of reported cases and the limited range of variants mean that the phenotypic profile of NSF-related disorders is incomplete. Secondly, the functional consequences of the identified variant were not directly assessed, so further functional investigations are needed, such as protein expression analyses and cellular models. Thirdly, while the identified NSF variant offers a plausible explanation for the patient’s phenotype, other molecular mechanisms, including genomic polymorphisms, epigenetic alterations and regulatory factors that may influence clinical expressivity, were not explored (Tüysüz et al., 2023; Coskunpinar et al., 2021). Fourthly, our trio-WES pipeline was designed and validated to detect SNVs and small indels within coding exons and flanking splice-site regions (±10 bp). However, it was not optimised to detect CNVs, structural rearrangements, chromosomal aneuploidies or mosaic variants. Future studies integrating functional validation with multi-omic analyses are needed to improve our understanding of NSF-DEE96.

In summary, our study reports a novel *de novo NSF* variant p (Asn352Ser) in a foetus with DEE96, broadening the disorder’s phenotype to include severe prenatal systemic manifestations such as anaemia, increased nuchal fold thickness and clubfoot. This study extends the genotype-phenotype spectrum of the disorder. Furthermore, our findings and literature review confirm DEE96 as a ‘SNAREopathy’, in which pathogenic variants disrupt vesicular fusion and autophagy, potentially via mTOR overactivation. This broadens our understanding of DEE96, shifting the focus from a purely neurodevelopmental disorder to a condition with multisystem fetal onset. Anemia emerges may be a key feature, emphasising the importance of considering NSF in the prenatal diagnosis of complex syndromes.

## Data Availability

The datasets presented in this study can be found in online repositories. The names of the repository/repositories and accession number(s) can be found at: https://www.ncbi.nlm.nih.gov/sra/PRJNA1492294.

## Supplementary material

SUPPLEMENTARY FIGURE S1 IGV plots of SNVs. Whole exome sequencing of NSF indicated the missense c.1055A>G; p.(Asn352Ser) variant was *de novo*.

## References

[B1] Baris S. Dogan M. Terali K. Gezdirici A. Eroz R. Yucel P. P. (2026). Biallelic truncating *DNAH14* variant in siblings with neurodevelopmental disorder and predominant ataxia: clinical report and literature review. Int. J. Mol. Sci. 27 (2), 575. 10.3390/ijms27020575 41596228 PMC12841059

[B2] Baudot A. D. Wang V. M. Leach J. D. O'Prey J. Long J. S. Paulus-Hock V. (2022). Glycan degradation promotes macroautophagy. Proc. Natl. Acad. Sci. U. S. A. 119 (26), e2111506119. 10.1073/pnas.2111506119 35737835 PMC9245654

[B3] Boycott K. M. Vanstone M. R. Bulman D. E. MacKenzie A. E. (2013). Rare-disease genetics in the era of next-generation sequencing: discovery to translation. Nat. Rev. Genet. 14 (10), 681–691. 10.1038/nrg3555 23999272

[B4] Chen M. Sandoval H. Wang J. (2008). Selective mitochondrial autophagy during erythroid maturation. Autophagy 4 (7), 926–928. 10.4161/auto.6716 18716457 PMC2900926

[B5] Coleman S. S. M. D. (1974). The incomplete pericapsular (pemberton) and innominate (salter) osteotomies: a complete analysis. Clin. Orthop. Relat. Res. 98, 116–123. 10.1097/00003086-197401000-00012 4817220

[B6] Coskunpinar E. Kose T. Algedik Demirayak P. Hayretdag C. Bozlak S. (2021). Investigation of VDR gene polymorphisms in twins with autism spectrum disorder. Res. Autism Spectr. Disord. 82, 101737. 10.1016/j.rasd.2021.101737

[B7] Ercoskun P. Akbulut E. Yavas C. Yilmaz Celik L. Dogan M. (2026). Delineating the adult phenotype of PGM2L1 -Related neurodevelopmental disorder. Am. J. Med. Genet. A 200 (3), 673–684. 10.1002/ajmg.a.64293 41178743

[B8] Ferro-Novick S. Brose N. (2013). Nobel 2013 physiology or medicine: traffic control system within cells. Nature 504 (7478), 98. 10.1038/504098a 24305158

[B9] Grosso R. Fader C. M. Colombo M. I. (2017). Autophagy: a necessary event during erythropoiesis. Blood Rev. 31 (5), 300–305. 10.1016/j.blre.2017.04.001 28483400

[B10] Hayashi T. Yano N. Kora K. Yokoyama A. Maizuru K. Kayaki T. (2023). Involvement of mTOR pathway in neurodegeneration in NSF-related developmental and epileptic encephalopathy. Hum. Mol. Genet. 32 (10), 1683–1697. 10.1093/hmg/ddad008 36645181 PMC10162430

[B11] Khan Y. A. White K. I. Brunger A. T. (2022). The AAA+ superfamily: a review of the structural and mechanistic principles of these molecular machines. Crit. Rev. Biochem. Mol. Biol. 57 (2), 156–187. 10.1080/10409238.2021.1979460 34632886

[B12] Khan Y. A. White K. I. Pfuetzner R. A. Singal B. Esquivies L. Mckenzie G. (2025). SNARE disassembly requires Sec18/NSF side loading. Nat. Struct. Mol. Biol. 32, 1708–1720. 10.1038/s41594-025-01590-w 40604310 PMC12440825

[B13] Levine B. Kroemer G. (2019). Biological functions of autophagy genes: a disease perspective. Cell. 176 (1-2), 11–42. 10.1016/j.cell.2018.09.048 30633901 PMC6347410

[B14] Li R. Chen G. L. Zhang Y. L. Li D. Z. (2024). NSF-related fetal anemia and hydrops: new entity? Ultrasound Obstet. Gynecol. 63 (3), 421–422. 10.1002/uog.27500 37767567

[B15] Mo W. Nicolson T. (2011). Both pre- and postsynaptic activity of Nsf prevents degeneration of hair-cell synapses. PLoS One 6 (11), e27146. 10.1371/journal.pone.0027146 22073277 PMC3207842

[B16] Moeller A. Zhao C. Fried M. G. Wilson-Kubalek E. M. Carragher B. Whiteheart S. W. (2012). Nucleotide-dependent conformational changes in the N-Ethylmaleimide sensitive factor (NSF) and their potential role in SNARE complex disassembly. J. Struct. Biol. 177 (2), 335–343. 10.1016/j.jsb.2011.12.018 22245547 PMC3382979

[B17] Morgan A. (1996). Classic clues to NSF function. Nature 382 (6593), 680. 10.1038/382680a0 8751437

[B18] Ng S. B. Buckingham K. J. Lee C. Bigham A. W. Tabor H. K. Dent K. M. (2010). Exome sequencing identifies the cause of a mendelian disorder. Nat. Genet. 42 (1), 30–35. 10.1038/ng.499 19915526 PMC2847889

[B19] Orr A. Song H. Rusin S. F. Kettenbach A. N. Wickner W. (2017). HOPS catalyzes the interdependent assembly of each vacuolar SNARE into a SNARE complex. Mol. Biol. Cell. 28 (7), 975–983. 10.1091/mbc.E16-10-0743 28148647 PMC5385945

[B20] Pallanck L. Ordway R. Ganetzky B. A. (1995). Drosophila NSF mutant. Nature 376, 25. 10.1038/376025a0 7596428

[B21] Ramakrishnan N. A. Drescher M. J. Drescher D. G. (2012). The SNARE complex in neuronal and sensory cells. Mol. Cell. Neurosci. 50 (1), 58–69. 10.1016/j.mcn.2012.03.009 22498053 PMC3570063

[B22] Richards S. Aziz N. Bale S. Bick D. Das S. Gastier-Foster J. (2015). Standards and guidelines for the interpretation of sequence variants: a joint consensus recommendation of the American college of medical genetics and genomics and the association for molecular pathology. Genet. Med. 17, 405–424. 10.1038/gim.2015.30 25741868 PMC4544753

[B23] Rizo J. (2018). Mechanism of neurotransmitter release coming into focus. Protein Sci. 27 (8), 1364–1391. 10.1002/pro.3445 29893445 PMC6153415

[B24] Saxton R. A. Sabatini D. M. (2017). mTOR signaling in growth, metabolism, and disease. Cell. 168(6):960–976. 10.1016/j.cell.2017.02.004 28283069 PMC5394987

[B25] Siddiqi O. Benzer S. (1976). Neurophysiological defects in temperature-sensitive paralytic mutants of *Drosophila melanogaster* . Proc. Natl. Acad. Sci. U.S.A. 73 (9), 3253–3257. 10.1073/pnas.73.9.3253 184469 PMC430996

[B26] Söllner T. Bennett M. K. Whiteheart S. W. Scheller R. H. Rothman J. E. (1993). A protein assembly-disassembly pathway *in vitro* that may correspond to sequential steps of synaptic vesicle docking, activation, and fusion. Cell. 75 (3), 409–418. 10.1016/0092-8674(93)90376-2 8221884

[B27] Stewart B. A. Mohtashami M. Rivlin P. Deitcher D. L. Trimble W. S. Boulianne G. L. (2002). Dominant-negative NSF2 disrupts the structure and function of drosophila neuromuscular synapses. J. Neurobiol. 51, 261–271. 10.1002/neu.10059 12150502

[B28] Suzuki H. Yoshida T. Morisada N. Uehara T. Kosaki K. Sato K. (2019). *De novo* NSF mutations cause early infantile epileptic encephalopathy. Ann. Clin. Transl. Neurol. 6 (11), 2334–2339. 10.1002/acn3.50917 31675180 PMC6856629

[B29] Tengsujaritkul M. Louthrenoo O. Likhitweerawong N. Boonchooduang N. Srisurapanont M. (2026). Diagnostic and clinical utility of exome sequencing and chromosomal microarray in children with GDD/iD: a meta-analysis. Ann. Med. 58 (1), 2609424. 10.1080/07853890.2025.2609424 41472336 PMC12777888

[B30] Tüysüz B. Bozlak S. Uludağ Alkaya D. Ocak S. Kasap B. Sunamak Çifçi E. (2023). Investigation of 11p15.5 methylation defects associated with beckwith-wiedemann spectrum and embryonic tumor risk in lateralized overgrowth patients. Cancers (Basel) 15 (6), 1872. 10.3390/cancers15061872 36980758 PMC10046725

[B31] White K. I. Zhao M. Choi U. B. Pfuetzner R. A. Brunger A. T. (2018). Structural principles of SNARE complex recognition by the AAA+ protein NSF eLife 7:e38888. 30198481 10.7554/eLife.38888 PMC6160233

[B32] White K. I. Khan Y. A. Qiu K. Balaji A. Couoh-Cardel S. Esquivies L. (2025). Structural remodeling of target-SNARE protein complexes by NSF enables synaptic transmission. Nat. Commun. 16, 8371. 10.1038/s41467-025-62764-0 40993127 PMC12460809

[B33] Woodman P. G. (1997). The roles of NSF, SNAPs and SNAREs during membrane fusion. Biochim. Biophys. Acta 1357 (2), 155–172. 10.1016/s0167-4889(97)00039-6 9223620

[B34] Yang Y. Muzny D. M. Reid J. G. Bainbridge M. N. Willis A. Ward P. A. (2013). Clinical whole-exome sequencing for the diagnosis of mendelian disorders. N. Engl. J. Med. 369 (16), 1502–1511. 10.1056/NEJMoa1306555 24088041 PMC4211433

[B35] Yavas C. Abuaisha A. Nekay E. Gezdirici A. Yilmaz H. I. Akbulut E. (2026). The role of ATP9A (c.1091G > C; p.(Arg364Thr)) variant in cognitive impairment: diagnostic insight from whole exome sequencing. Mol. Biol. Rep. 53 (1), 329. 10.1007/s11033-026-11496-5 41604004

[B36] Zhao M. Brunger A. T. (2016). Recent advances in deciphering the structure and molecular mechanism of the AAA+ ATPase N-Ethylmaleimide-Sensitive factor (NSF). J. Mol. Biol. 428 (9 Pt B), 1912–1926. 10.1016/j.jmb.2015.10.026 26546278 PMC4854814

[B37] Zhao C. Smith E. C. Whiteheart S. W. (2012). Requirements for the catalytic cycle of the N-ethylmaleimide-Sensitive factor (NSF). Biochim. Biophys. Acta 1823 (1), 159–171. 10.1016/j.bbamcr.2011.06.003 21689688 PMC3983028

